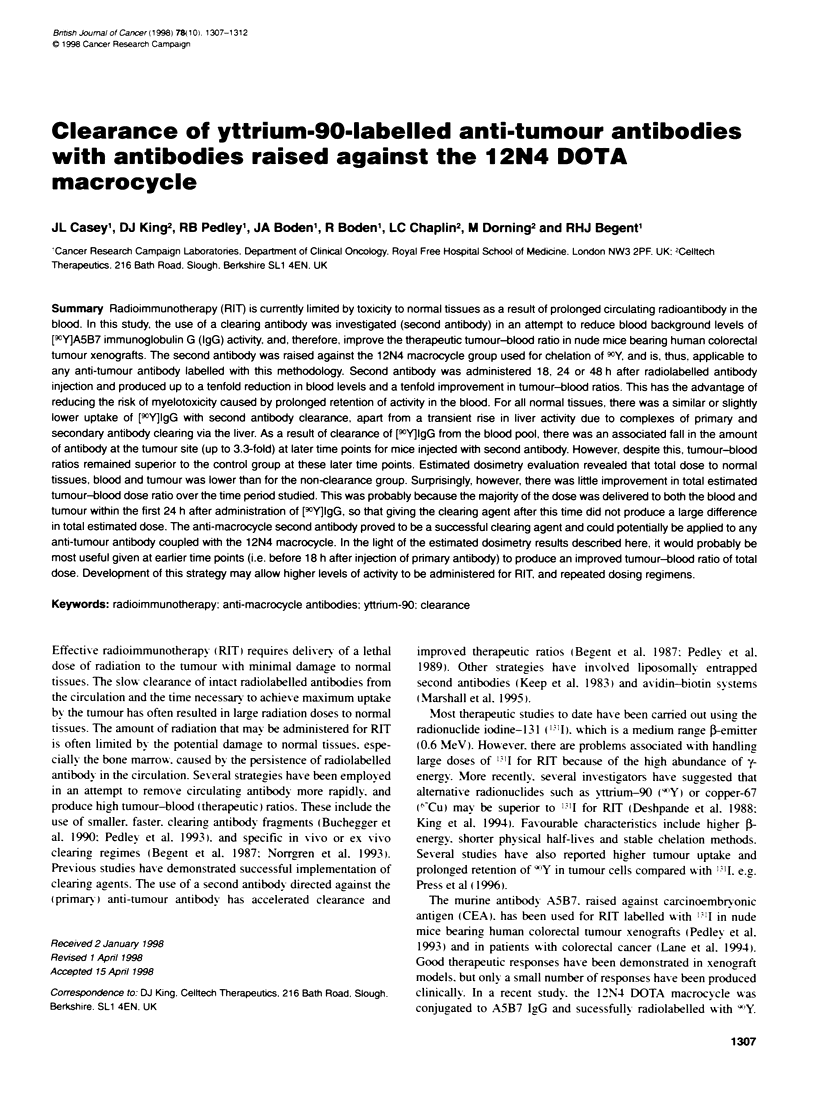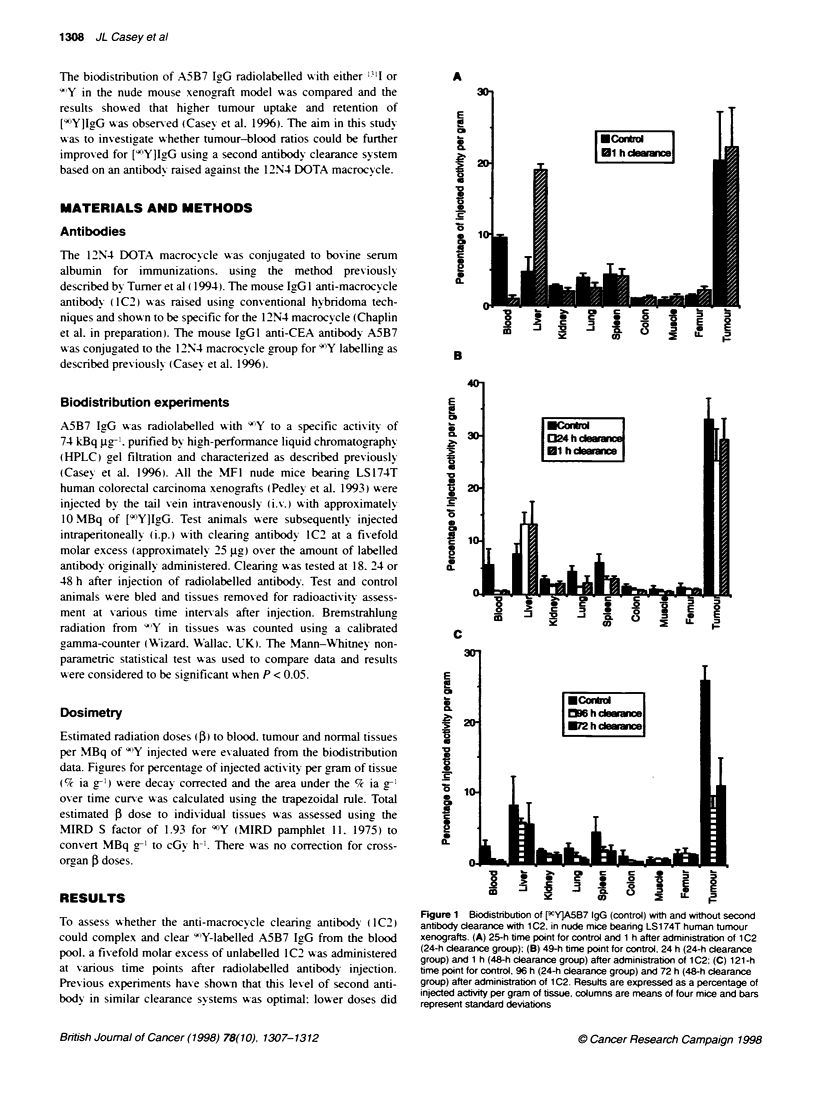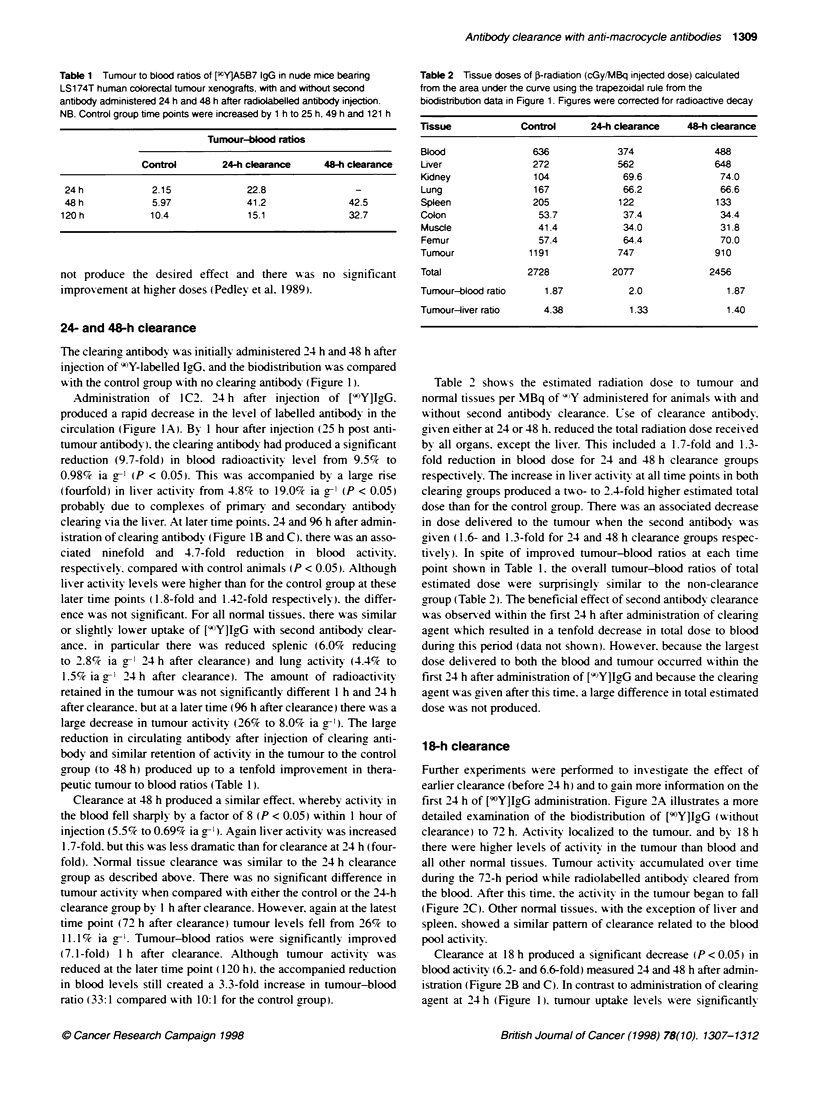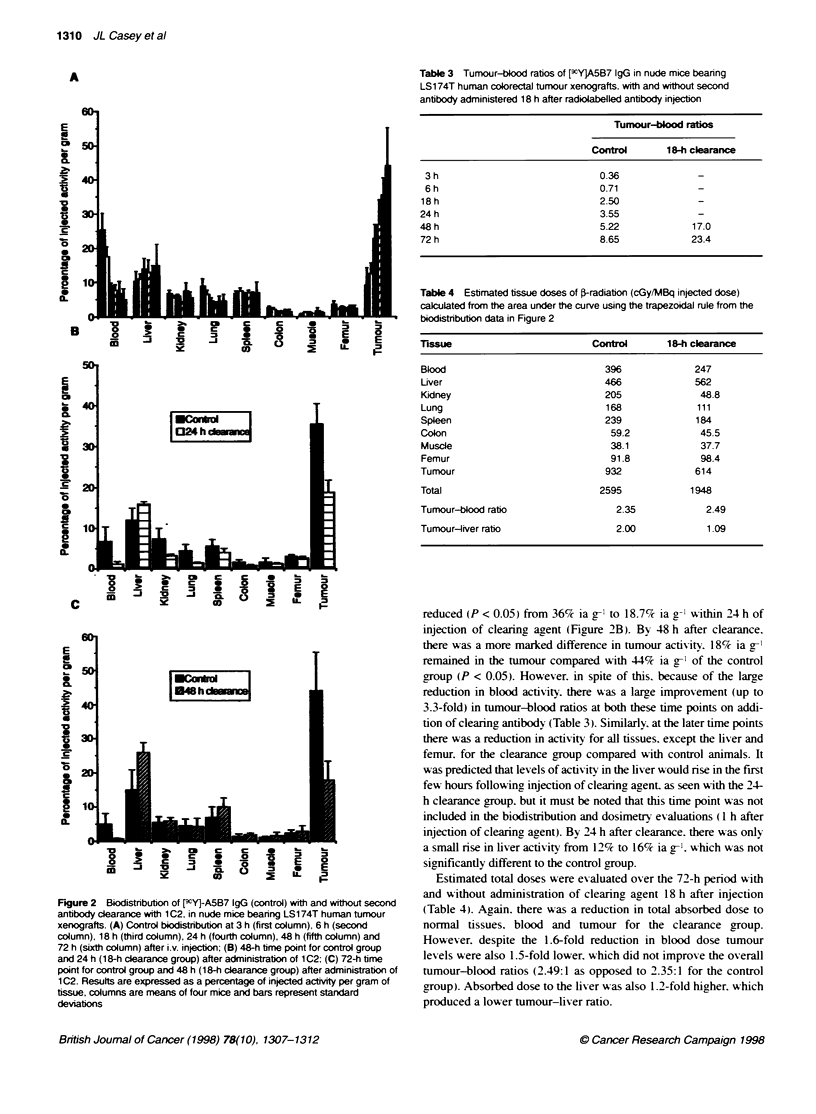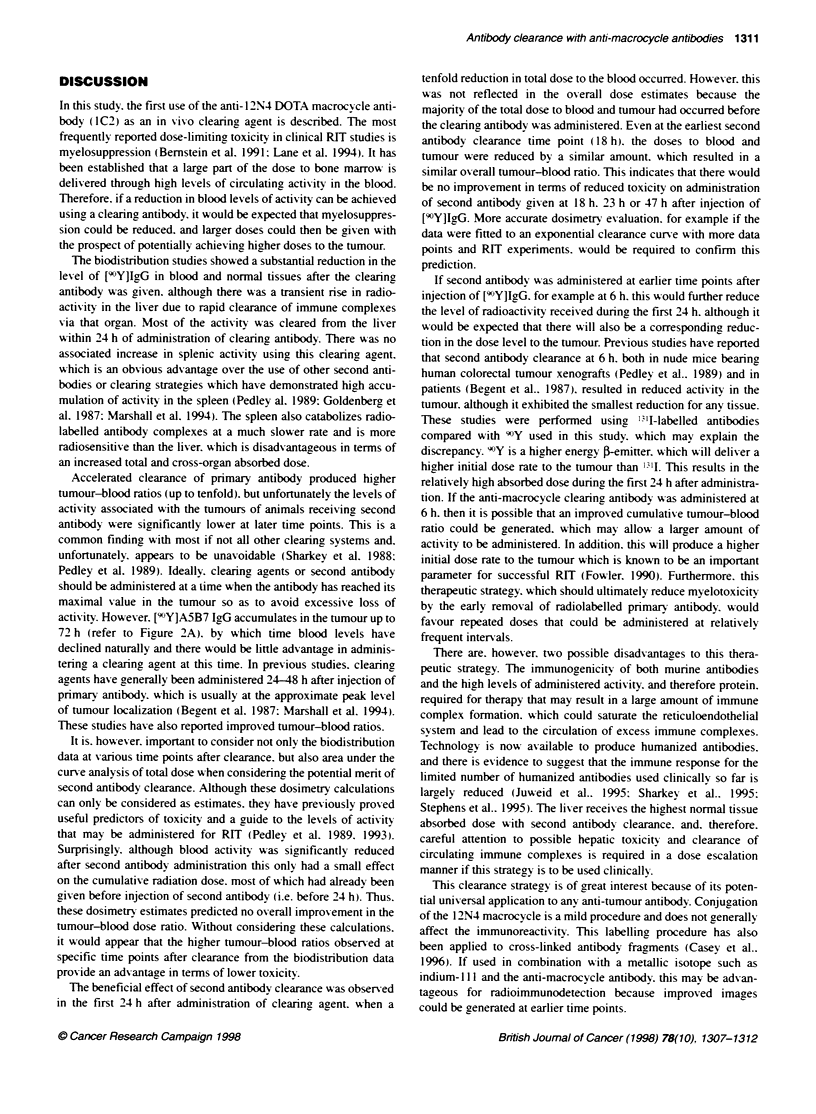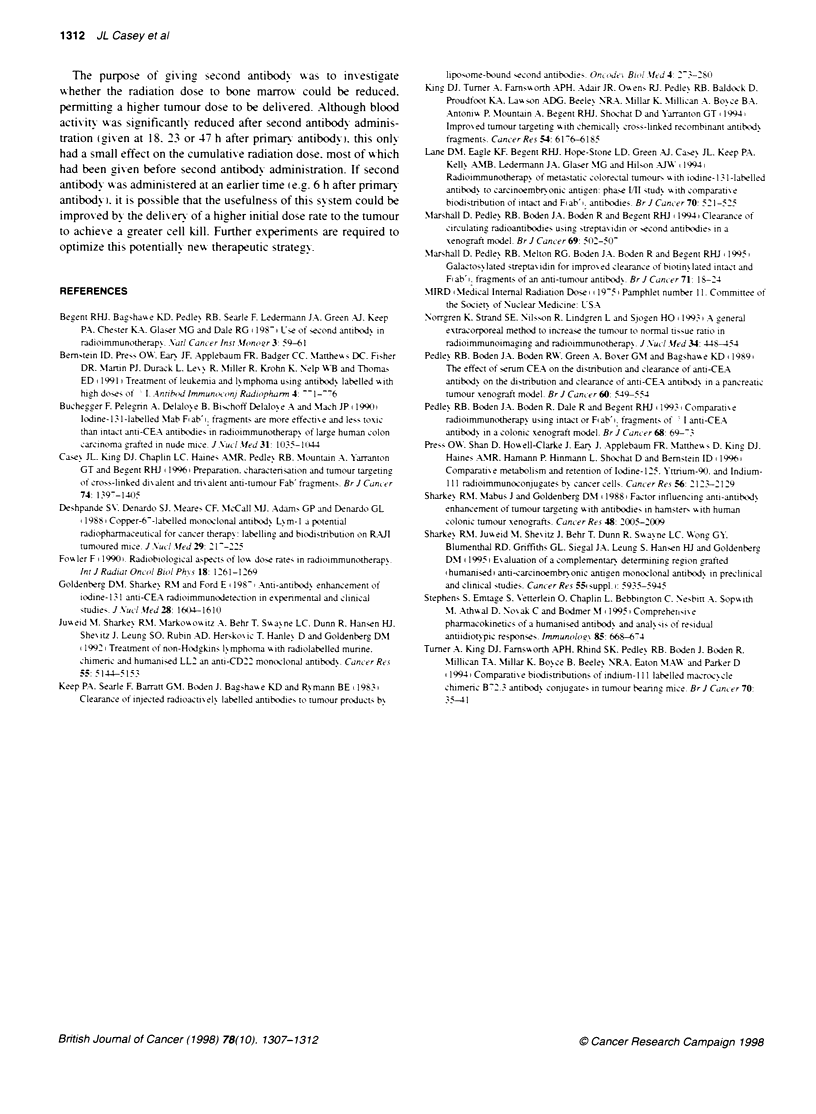# Clearance of yttrium-90-labelled anti-tumour antibodies with antibodies raised against the 12N4 DOTA macrocycle.

**DOI:** 10.1038/bjc.1998.676

**Published:** 1998-11

**Authors:** J. L. Casey, D. J. King, R. B. Pedley, J. A. Boden, R. Boden, L. C. Chaplin, M. Dorning, R. H. Begent

**Affiliations:** Department of Clinical Oncology, Royal Free Hospital School of Medicine, London, UK.

## Abstract

Radioimmunotherapy (RIT) is currently limited by toxicity to normal tissues as a result of prolonged circulating radioantibody in the blood. In this study, the use of a clearing antibody was investigated (second antibody) in an attempt to reduce blood background levels of [90Y]A5B7 immunoglobulin G (IgG) activity, and, therefore, improve the therapeutic tumour-blood ratio in nude mice bearing human colorectal tumour xenografts. The second antibody was raised against the 12N4 macrocycle group used for chelation of 90Y, and is, thus, applicable to any anti-tumour antibody labelled with this methodology. Second antibody was administered 18, 24 or 48 h after radiolabelled antibody injection and produced up to a tenfold reduction in blood levels and a tenfold improvement in tumour-blood ratios. This has the advantage of reducing the risk of myelotoxicity caused by prolonged retention of activity in the blood. For all normal tissues, there was a similar or slightly lower uptake of [90Y]IgG with second antibody clearance, apart from a transient rise in liver activity due to complexes of primary and secondary antibody clearing via the liver. As a result of clearance of [90Y]IgG from the blood pool, there was an associated fall in the amount of antibody at the tumour site (up to 3.3-fold) at later time points for mice injected with second antibody. However, despite this, tumour-blood ratios remained superior to the control group at these later time points. Estimated dosimetry evaluation revealed that total dose to normal tissues, blood and tumour was lower than for the non-clearance group. Surprisingly, however, there was little improvement in total estimated tumour-blood dose ratio over the time period studied. This was probably because the majority of the dose was delivered to both the blood and tumour within the first 24 h after administration of [90Y]IgG, so that giving the clearing agent after this time did not produce a large difference in total estimated dose. The anti-macrocycle second antibody proved to be a successful clearing agent and could potentially be applied to any anti-tumour antibody coupled with the 12N4 macrocycle. In the light of the estimated dosimetry results described here, it would probably be most useful given at earlier time points (i.e. before 18 h after injection of primary antibody) to produce an improved tumour-blood ratio of total dose. Development of this strategy may allow higher levels of activity to be administered for RIT, and repeated dosing regimens.


British Journal of Cancer (1998) 78(10), 1307–1312
© 1998 Cancer Research Campaign

### Clearance of yttrium-90-labelled anti-tumour antibodies with antibodies raised against the 12N4 DOTA macrocycle

**JL Casey[1], DJ King[2], RB Pedley[1], JA Boden[1], R Boden[1], LC Chaplin[2], M Dorning[2] and RHJ Begent[1]**

[1]Cancer Research Campaign Laboratories, Department of Clinical Oncology, Royal Free Hospital School of Medicine, London NW3 2PF, UK; [2]Celltech Therapeutics, 216 Bath Road, Slough, Berkshire SL1 4EN, UK

**Summary** Radioimmunotherapy (RIT) is currently limited by toxicity to normal tissues as a result of prolonged circulating radioantibody in the blood. In this study, the use of a clearing antibody was investigated (second antibody) in an attempt to reduce blood background levels of [$^{90}$Y]A5B7 immunoglobulin G (IgG) activity, and, therefore, improve the therapeutic tumour–blood ratio in nude mice bearing human colorectal tumour xenografts. The second antibody was raised against the 12N4 macrocycle group used for chelation of $^{90}$Y, and is, thus, applicable to any anti-tumour antibody labelled with this methodology. Second antibody was administered 18, 24 or 48 h after radiolabelled antibody injection and produced up to a tenfold reduction in blood levels and a tenfold improvement in tumour–blood ratios. This has the advantage of reducing the risk of myelotoxicity caused by prolonged retention of activity in the blood. For all normal tissues, there was a similar or slightly lower uptake of [$^{90}$Y]IgG with second antibody clearance, apart from a transient rise in liver activity due to complexes of primary and secondary antibody clearing via the liver. As a result of clearance of [$^{90}$Y]IgG from the blood pool, there was an associated fall in the amount of antibody at the tumour site (up to 3.3-fold) at later time points for mice injected with second antibody. However, despite this, tumour–blood ratios remained superior to the control group at these later time points. Estimated dosimetry evaluation revealed that total dose to normal tissues, blood and tumour was lower than for the non-clearance group. Surprisingly, however, there was little improvement in total estimated tumour–blood dose ratio over the time period studied. This was probably because the majority of the dose was delivered to both the blood and tumour within the first 24 h after administration of [$^{90}$Y]IgG, so that giving the clearing agent after this time did not produce a large difference in total estimated dose. The anti-macrocycle second antibody proved to be a successful clearing agent and could potentially be applied to any anti-tumour antibody coupled with the 12N4 macrocycle. In the light of the estimated dosimetry results described here, it would probably be most useful given at earlier time points (i.e. before 18 h after injection of primary antibody) to produce an improved tumour–blood ratio of total dose. Development of this strategy may allow higher levels of activity to be administered for RIT, and repeated dosing regimens.

**Keywords:** radioimmunotherapy; anti-macrocycle antibodies; yttrium-90; clearance

Effective radioimmunotherapy (RIT) requires delivery of a lethal dose of radiation to the tumour with minimal damage to normal tissues. The slow clearance of intact radiolabelled antibodies from the circulation and the time necessary to achieve maximum uptake by the tumour has often resulted in large radiation doses to normal tissues. The amount of radiation that may be administered for RIT is often limited by the potential damage to normal tissues, especially the bone marrow, caused by the persistence of radiolabelled antibody in the circulation. Several strategies have been employed in an attempt to remove circulating antibody more rapidly, and produce high tumour–blood (therapeutic) ratios. These include the use of smaller, faster, clearing antibody fragments (Buchegger et al. 1990; Pedley et al. 1993), and specific in vivo or ex vivo clearing regimes (Begent et al. 1987; Norrgren et al. 1993). Previous studies have demonstrated successful implementation of clearing agents. The use of a second antibody directed against the (primary) anti-tumour antibody has accelerated clearance and improved therapeutic ratios (Begent et al. 1987; Pedley et al. 1989). Other strategies have involved liposomally entrapped second antibodies (Keep et al. 1983) and avidin–biotin systems (Marshall et al. 1995).

*Received 2 January 1998*
*Revised 1 April 1998*
*Accepted 15 April 1998*

*Correspondence to:* DJ King, Celltech Therapeutics, 216 Bath Road, Slough, Berkshire, SL1 4EN, UK

Most therapeutic studies to date have been carried out using the radionuclide iodine-131 ($^{131}$I), which is a medium range β-emitter (0.6 MeV). However, there are problems associated with handling large doses of $^{131}$I for RIT because of the high abundance of γ-energy. More recently, several investigators have suggested that alternative radionuclides such as yttrium-90 ($^{90}$Y) or copper-67 ($^{67}$Cu) may be superior to $^{131}$I for RIT (Deshpande et al. 1988; King et al. 1994). Favourable characteristics include higher β-energy, shorter physical half-lives and stable chelation methods. Several studies have also reported higher tumour uptake and prolonged retention of $^{90}$Y in tumour cells compared with $^{131}$I, e.g. Press et al (1996).

The murine antibody A5B7, raised against carcinoembryonic antigen (CEA), has been used for RIT labelled with $^{131}$I in nude mice bearing human colorectal tumour xenografts (Pedley et al. 1993) and in patients with colorectal cancer (Lane et al. 1994). Good therapeutic responses have been demonstrated in xenograft models, but only a small number of responses have been produced clinically. In a recent study, the 12N4 DOTA macrocycle was conjugated to A5B7 IgG and sucessfully radiolabelled with $^{90}$Y.

The biodistribution of A5B7 IgG radiolabelled with either $^{131}$I or $^{90}$Y in the nude mouse xenograft model was compared and the results showed that higher tumour uptake and retention of [$^{90}$Y]IgG was observed (Casey et al. 1996). The aim in this study was to investigate whether tumour–blood ratios could be further improved for [$^{90}$Y]IgG using a second antibody clearance system based on an antibody raised against the 12N4 DOTA macrocycle.

### MATERIALS AND METHODS

### Antibodies

The 12N4 DOTA macrocycle was conjugated to bovine serum albumin for immunizations, using the method previously described by Turner et al (1994). The mouse IgG1 anti-macrocycle antibody (1C2) was raised using conventional hybridoma techniques and shown to be specific for the 12N4 macrocycle (Chaplin et al. in preparation). The mouse IgG1 anti-CEA antibody A5B7 was conjugated to the 12N4 macrocycle group for $^{90}$Y labelling as described previously (Casey et al. 1996).

### Biodistribution experiments

A5B7 IgG was radiolabelled with $^{90}$Y to a specific activity of 74 kBq $\mu g^{-1}$, purified by high-performance liquid chromatography (HPLC) gel filtration and characterized as described previously (Casey et al. 1996). All the MF1 nude mice bearing LS174T human colorectal carcinoma xenografts (Pedley et al. 1993) were injected by the tail vein intravenously (i.v.) with approximately 10 MBq of [$^{90}$Y]IgG. Test animals were subsequently injected intraperitoneally (i.p.) with clearing antibody 1C2 at a fivefold molar excess (approximately 25 µg) over the amount of labelled antibody originally administered. Clearing was tested at 18, 24 or 48 h after injection of radiolabelled antibody. Test and control animals were bled and tissues removed for radioactivity assessment at various time intervals after injection. Bremstrahlung radiation from $^{90}$Y in tissues was counted using a calibrated gamma-counter (Wizard, Wallac, UK). The Mann–Whitney non-parametric statistical test was used to compare data and results were considered to be significant when $P < 0.05$.

### Dosimetry

Estimated radiation doses (β) to blood, tumour and normal tissues per MBq of $^{90}$Y injected were evaluated from the biodistribution data. Figures for percentage of injected activity per gram of tissue (% ia $g^{-1}$) were decay corrected and the area under the % ia $g^{-1}$ over time curve was calculated using the trapezoidal rule. Total estimated β dose to individual tissues was assessed using the MIRD S factor of 1.93 for $^{90}$Y (MIRD pamphlet 11, 1975) to convert MBq $g^{-1}$ to cGy $h^{-1}$. There was no correction for cross-organ β doses.

### RESULTS

To assess whether the anti-macrocycle clearing antibody (1C2) could complex and clear $^{90}$Y-labelled A5B7 IgG from the blood pool, a fivefold molar excess of unlabelled 1C2 was administered at various time points after radiolabelled antibody injection. Previous experiments have shown that this level of second antibody in similar clearance systems was optimal: lower doses did

**Figure 1** Biodistribution of [$^{90}$Y]A5B7 IgG (control) with and without second antibody clearance with 1C2, in nude mice bearing LS174T human tumour xenografts. (**A**) 25-h time point for control and 1 h after administration of 1C2 (24-h clearance group); (**B**) 49-h time point for control, 24 h (24-h clearance group) and 1 h (48-h clearance group) after administration of 1C2; (**C**) 121-h time point for control, 96 h (24-h clearance group) and 72 h (48-h clearance group) after administration of 1C2. Results are expressed as a percentage of injected activity per gram of tissue, columns are means of four mice and bars represent standard deviations

 *© Cancer Research Campaign 1998*

**Table 1** Tumour to blood ratios of [$^{90}$Y]A5B7 IgG in nude mice bearing LS174T human colorectal tumour xenografts, with and without second antibody administered 24 h and 48 h after radiolabelled antibody injection. NB. Control group time points were increased by 1 h to 25 h, 49 h and 121 h

| | Tumour–blood ratios | | |
|---|---|---|---|
| | **Control** | **24-h clearance** | **48-h clearance** |
| 24 h | 2.15 | 22.8 | – |
| 48 h | 5.97 | 41.2 | 42.5 |
| 120 h | 10.4 | 15.1 | 32.7 |

not produce the desired effect and there was no significant improvement at higher doses (Pedley et al, 1989).

### 24- and 48-h clearance

The clearing antibody was initially administered 24 h and 48 h after injection of $^{90}$Y-labelled IgG, and the biodistribution was compared with the control group with no clearing antibody (Figure 1).

Administration of 1C2, 24 h after injection of [$^{90}$Y]IgG, produced a rapid decrease in the level of labelled antibody in the circulation (Figure 1A). By 1 hour after injection (25 h post anti-tumour antibody), the clearing antibody had produced a significant reduction (9.7-fold) in blood radioactivity level from 9.5% to 0.98% ia $g^{-1}$ ($P < 0.05$). This was accompanied by a large rise (fourfold) in liver activity from 4.8% to 19.0% ia $g^{-1}$ ($P < 0.05$) probably due to complexes of primary and secondary antibody clearing via the liver. At later time points, 24 and 96 h after administration of clearing antibody (Figure 1B and C), there was an associated ninefold and 4.7-fold reduction in blood activity, respectively, compared with control animals ($P < 0.05$). Although liver activity levels were higher than for the control group at these later time points (1.8-fold and 1.42-fold respectively), the difference was not significant. For all normal tissues, there was similar or slightly lower uptake of [$^{90}$Y]IgG with second antibody clearance, in particular there was reduced splenic (6.0% reducing to 2.8% ia $g^{-1}$ 24 h after clearance) and lung activity (4.4% to 1.5% ia $g^{-1}$ 24 h after clearance). The amount of radioactivity retained in the tumour was not significantly different 1 h and 24 h after clearance, but at a later time (96 h after clearance) there was a large decrease in tumour activity (26% to 8.0% ia $g^{-1}$). The large reduction in circulating antibody after injection of clearing antibody and similar retention of activity in the tumour to the control group (to 48 h) produced up to a tenfold improvement in therapeutic tumour to blood ratios (Table 1).

Clearance at 48 h produced a similar effect, whereby activity in the blood fell sharply by a factor of 8 ($P < 0.05$) within 1 hour of injection (5.5% to 0.69% ia $g^{-1}$). Again liver activity was increased 1.7-fold, but this was less dramatic than for clearance at 24 h (fourfold). Normal tissue clearance was similar to the 24 h clearance group as described above. There was no significant difference in tumour activity when compared with either the control or the 24-h clearance group by 1 h after clearance. However, again at the latest time point (72 h after clearance) tumour levels fell from 26% to 11.1% ia $g^{-1}$. Tumour–blood ratios were significantly improved (7.1-fold) 1 h after clearance. Although tumour activity was reduced at the later time point (120 h), the accompanied reduction in blood levels still created a 3.3-fold increase in tumour–blood ratio (33:1 compared with 10:1 for the control group).

**Table 2** Tissue doses of β-radiation (cGy/MBq injected dose) calculated from the area under the curve using the trapezoidal rule from the biodistribution data in Figure 1. Figures were corrected for radioactive decay

| Tissue | Control | 24-h clearance | 48-h clearance |
|---|---|---|---|
| Blood | 636 | 374 | 488 |
| Liver | 272 | 562 | 648 |
| Kidney | 104 | 69.6 | 74.0 |
| Lung | 167 | 66.2 | 66.6 |
| Spleen | 205 | 122 | 133 |
| Colon | 53.7 | 37.4 | 34.4 |
| Muscle | 41.4 | 34.0 | 31.8 |
| Femur | 57.4 | 64.4 | 70.0 |
| Tumour | 1191 | 747 | 910 |
| Total | 2728 | 2077 | 2456 |
| Tumour–blood ratio | 1.87 | 2.0 | 1.87 |
| Tumour–liver ratio | 4.38 | 1.33 | 1.40 |

Table 2 shows the estimated radiation dose to tumour and normal tissues per MBq of $^{90}$Y administered for animals with and without second antibody clearance. Use of clearance antibody, given either at 24 or 48 h, reduced the total radiation dose received by all organs, except the liver. This included a 1.7-fold and 1.3-fold reduction in blood dose for 24 and 48 h clearance groups respectively. The increase in liver activity at all time points in both clearing groups produced a two- to 2.4-fold higher estimated total dose than for the control group. There was an associated decrease in dose delivered to the tumour when the second antibody was given (1.6- and 1.3-fold for 24 and 48 h clearance groups respectively). In spite of improved tumour–blood ratios at each time point shown in Table 1, the overall tumour–blood ratios of total estimated dose were surprisingly similar to the non-clearance group (Table 2). The beneficial effect of second antibody clearance was observed within the first 24 h after administration of clearing agent which resulted in a tenfold decrease in total dose to blood during this period (data not shown). However, because the largest dose delivered to both the blood and tumour occurred within the first 24 h after administration of [$^{90}$Y]IgG and because the clearing agent was given after this time, a large difference in total estimated dose was not produced.

### 18-h clearance

Further experiments were performed to investigate the effect of earlier clearance (before 24 h) and to gain more information on the first 24 h of [$^{90}$Y]IgG administration. Figure 2A illustrates a more detailed examination of the biodistribution of [$^{90}$Y]IgG (without clearance) to 72 h. Activity localized to the tumour, and by 18 h there were higher levels of activity in the tumour than blood and all other normal tissues. Tumour activity accumulated over time during the 72-h period while radiolabelled antibody cleared from the blood. After this time, the activity in the tumour began to fall (Figure 2C). Other normal tissues, with the exception of liver and spleen, showed a similar pattern of clearance related to the blood pool activity.

Clearance at 18 h produced a significant decrease ($P < 0.05$) in blood activity (6.2- and 6.6-fold) measured 24 and 48 h after administration (Figure 2B and C). In contrast to administration of clearing agent at 24 h (Figure 1), tumour uptake levels were significantly

**Figure 2** Biodistribution of $[^{90}Y]$-A5B7 IgG (control) with and without second antibody clearance with 1C2, in nude mice bearing LS174T human tumour xenografts. (**A**) Control biodistribution at 3 h (first column), 6 h (second column), 18 h (third column), 24 h (fourth column), 48 h (fifth column) and 72 h (sixth column) after i.v. injection; (**B**) 48-h time point for control group and 24 h (18-h clearance group) after administration of 1C2; (**C**) 72-h time point for control group and 48 h (18-h clearance group) after administration of 1C2. Results are expressed as a percentage of injected activity per gram of tissue, columns are means of four mice and bars represent standard deviations

**Table 3** Tumour–blood ratios of $[^{90}Y]$A5B7 IgG in nude mice bearing LS174T human colorectal tumour xenografts, with and without second antibody administered 18 h after radiolabelled antibody injection

| | Tumour–blood ratios | |
|---|---|---|
| | **Control** | **18-h clearance** |
| 3 h | 0.36 | – |
| 6 h | 0.71 | – |
| 18 h | 2.50 | – |
| 24 h | 3.55 | – |
| 48 h | 5.22 | 17.0 |
| 72 h | 8.65 | 23.4 |

**Table 4** Estimated tissue doses of β-radiation (cGy/MBq injected dose) calculated from the area under the curve using the trapezoidal rule from the biodistribution data in Figure 2

| **Tissue** | **Control** | **18-h clearance** |
|---|---|---|
| Blood | 396 | 247 |
| Liver | 466 | 562 |
| Kidney | 205 | 48.8 |
| Lung | 168 | 111 |
| Spleen | 239 | 184 |
| Colon | 59.2 | 45.5 |
| Muscle | 38.1 | 37.7 |
| Femur | 91.8 | 98.4 |
| Tumour | 932 | 614 |
| Total | 2595 | 1948 |
| Tumour–blood ratio | 2.35 | 2.49 |
| Tumour–liver ratio | 2.00 | 1.09 |

reduced ($P < 0.05$) from 36% ia $g^{-1}$ to 18.7% ia $g^{-1}$ within 24 h of injection of clearing agent (Figure 2B). By 48 h after clearance, there was a more marked difference in tumour activity, 18% ia $g^{-1}$ remained in the tumour compared with 44% ia $g^{-1}$ of the control group ($P < 0.05$). However, in spite of this, because of the large reduction in blood activity, there was a large improvement (up to 3.3-fold) in tumour–blood ratios at both these time points on addition of clearing antibody (Table 3). Similarly, at the later time points there was a reduction in activity for all tissues, except the liver and femur, for the clearance group compared with control animals. It was predicted that levels of activity in the liver would rise in the first few hours following injection of clearing agent, as seen with the 24-h clearance group, but it must be noted that this time point was not included in the biodistribution and dosimetry evaluations (1 h after injection of clearing agent). By 24 h after clearance, there was only a small rise in liver activity from 12% to 16% ia $g^{-1}$, which was not significantly different to the control group.

Estimated total doses were evaluated over the 72-h period with and without administration of clearing agent 18 h after injection (Table 4). Again, there was a reduction in total absorbed dose to normal tissues, blood and tumour for the clearance group. However, despite the 1.6-fold reduction in blood dose tumour levels were also 1.5-fold lower, which did not improve the overall tumour–blood ratios (2.49:1 as opposed to 2.35:1 for the control group). Absorbed dose to the liver was also 1.2-fold higher, which produced a lower tumour–liver ratio.

*© Cancer Research Campaign 1998*

### DISCUSSION

In this study, the first use of the anti-12N4 DOTA macrocycle antibody (1C2) as an in vivo clearing agent is described. The most frequently reported dose-limiting toxicity in clinical RIT studies is myelosuppression (Bernstein et al, 1991; Lane et al, 1994). It has been established that a large part of the dose to bone marrow is delivered through high levels of circulating activity in the blood. Therefore, if a reduction in blood levels of activity can be achieved using a clearing antibody, it would be expected that myelosuppression could be reduced, and larger doses could then be given with the prospect of potentially achieving higher doses to the tumour.

The biodistribution studies showed a substantial reduction in the level of [$^{90}$Y]IgG in blood and normal tissues after the clearing antibody was given, although there was a transient rise in radioactivity in the liver due to rapid clearance of immune complexes via that organ. Most of the activity was cleared from the liver within 24 h of administration of clearing antibody. There was no associated increase in splenic activity using this clearing agent, which is an obvious advantage over the use of other second antibodies or clearing strategies which have demonstrated high accumulation of activity in the spleen (Pedley al, 1989; Goldenberg et al, 1987; Marshall et al, 1994). The spleen also catabolizes radiolabelled antibody complexes at a much slower rate and is more radiosensitive than the liver, which is disadvantageous in terms of an increased total and cross-organ absorbed dose.

Accelerated clearance of primary antibody produced higher tumour–blood ratios (up to tenfold), but unfortunately the levels of activity associated with the tumours of animals receiving second antibody were significantly lower at later time points. This is a common finding with most if not all other clearing systems and, unfortunately, appears to be unavoidable (Sharkey et al, 1988; Pedley et al, 1989). Ideally, clearing agents or second antibody should be administered at a time when the antibody has reached its maximal value in the tumour so as to avoid excessive loss of activity. However, [$^{90}$Y]A5B7 IgG accumulates in the tumour up to 72 h (refer to Figure 2A), by which time blood levels have declined naturally and there would be little advantage in administering a clearing agent at this time. In previous studies, clearing agents have generally been administered 24–48 h after injection of primary antibody, which is usually at the approximate peak level of tumour localization (Begent et al, 1987; Marshall et al, 1994). These studies have also reported improved tumour–blood ratios.

It is, however, important to consider not only the biodistribution data at various time points after clearance, but also area under the curve analysis of total dose when considering the potential merit of second antibody clearance. Although these dosimetry calculations can only be considered as estimates, they have previously proved useful predictors of toxicity and a guide to the levels of activity that may be administered for RIT (Pedley et al, 1989, 1993). Surprisingly, although blood activity was significantly reduced after second antibody administration this only had a small effect on the cumulative radiation dose, most of which had already been given before injection of second antibody (i.e. before 24 h). Thus, these dosimetry estimates predicted no overall improvement in the tumour–blood dose ratio. Without considering these calculations, it would appear that the higher tumour–blood ratios observed at specific time points after clearance from the biodistribution data provide an advantage in terms of lower toxicity.

The beneficial effect of second antibody clearance was observed in the first 24 h after administration of clearing agent, when a tenfold reduction in total dose to the blood occurred. However, this was not reflected in the overall dose estimates because the majority of the total dose to blood and tumour had occurred before the clearing antibody was administered. Even at the earliest second antibody clearance time point (18 h), the doses to blood and tumour were reduced by a similar amount, which resulted in a similar overall tumour–blood ratio. This indicates that there would be no improvement in terms of reduced toxicity on administration of second antibody given at 18 h, 23 h or 47 h after injection of [$^{90}$Y]IgG. More accurate dosimetry evaluation, for example if the data were fitted to an exponential clearance curve with more data points and RIT experiments, would be required to confirm this prediction.

If second antibody was administered at earlier time points after injection of [$^{90}$Y]IgG, for example at 6 h, this would further reduce the level of radioactivity received during the first 24 h, although it would be expected that there will also be a corresponding reduction in the dose level to the tumour. Previous studies have reported that second antibody clearance at 6 h, both in nude mice bearing human colorectal tumour xenografts (Pedley et al., 1989) and in patients (Begent et al., 1987), resulted in reduced activity in the tumour, although it exhibited the smallest reduction for any tissue. These studies were performed using $^{131}$I-labelled antibodies compared with $^{90}$Y used in this study, which may explain the discrepancy. $^{90}$Y is a higher energy β-emitter, which will deliver a higher initial dose rate to the tumour than $^{131}$I. This results in the relatively high absorbed dose during the first 24 h after administration. If the anti-macrocycle clearing antibody was administered at 6 h, then it is possible that an improved cumulative tumour–blood ratio could be generated, which may allow a larger amount of activity to be administered. In addition, this will produce a higher initial dose rate to the tumour which is known to be an important parameter for successful RIT (Fowler, 1990). Furthermore, this therapeutic strategy, which should ultimately reduce myelotoxicity by the early removal of radiolabelled primary antibody, would favour repeated doses that could be administered at relatively frequent intervals.

There are, however, two possible disadvantages to this therapeutic strategy. The immunogenicity of both murine antibodies and the high levels of administered activity, and therefore protein, required for therapy that may result in a large amount of immune complex formation, which could saturate the reticuloendothelial system and lead to the circulation of excess immune complexes. Technology is now available to produce humanized antibodies, and there is evidence to suggest that the immune response for the limited number of humanized antibodies used clinically so far is largely reduced (Juweid et al., 1995; Sharkey et al., 1995; Stephens et al., 1995). The liver receives the highest normal tissue absorbed dose with second antibody clearance, and, therefore, careful attention to possible hepatic toxicity and clearance of circulating immune complexes is required in a dose escalation manner if this strategy is to be used clinically.

This clearance strategy is of great interest because of its potential universal application to any anti-tumour antibody. Conjugation of the 12N4 macrocycle is a mild procedure and does not generally affect the immunoreactivity. This labelling procedure has also been applied to cross-linked antibody fragments (Casey et al., 1996). If used in combination with a metallic isotope such as indium-111 and the anti-macrocycle antibody, this may be advantageous for radioimmunodetection because improved images could be generated at earlier time points.

© Cancer Research Campaign 1998 

The purpose of giving second antibody was to investigate whether the radiation dose to bone marrow could be reduced, permitting a higher tumour dose to be delivered. Although blood activity was significantly reduced after second antibody administration (given at 18, 23 or 47 h after primary antibody), this only had a small effect on the cumulative radiation dose, most of which had been given before second antibody administration. If second antibody was administered at an earlier time (e.g. 6 h after primary antibody), it is possible that the usefulness of this system could be improved by the delivery of a higher initial dose rate to the tumour to achieve a greater cell kill. Further experiments are required to optimize this potentially new therapeutic strategy.

### REFERENCES

Begent RHJ, Bagshawe KD, Pedley RB, Searle F, Ledermann JA, Green AJ, Keep PA, Chester KA, Glaser MG and Dale RG (1987) Use of second antibody in radioimmunotherapy. *Natl Cancer Inst Monogr* **3**: 59–61

Bernstein ID, Press OW, Eary JF, Applebaum FR, Badger CC, Matthews DC, Fisher DR, Martin PJ, Durack L, Levy R, Miller R, Krohn K, Nelp WB and Thomas ED (1991) Treatment of leukemia and lymphoma using antibody labelled with high doses of [illegible]I. *Antibod Immunoconj Radiopharm* **4**: 771–776

Buchegger F, Pelegrin A, Delaloye B, Bischoff Delaloye A and Mach JP (1990) Iodine-131-labelled Mab F(ab')$_2$ fragments are more effective and less toxic than intact anti-CEA antibodies in radioimmunotherapy of large human colon carcinoma grafted in nude mice. *J Nucl Med* **31**: 1035–1044

Casey JL, King DJ, Chaplin LC, Haines AMR, Pedley RB, Mountain A, Yarranton GT and Begent RHJ (1996) Preparation, characterisation and tumour targeting of cross-linked divalent and trivalent anti-tumour Fab' fragments. *Br J Cancer* **74**: 1397–1405

Deshpande SV, Denardo SJ, Meares CF, McCall MJ, Adams GP and Denardo GL (1988) Copper-67-labelled monoclonal antibody Lym-1 a potential radiopharmaceutical for cancer therapy: labelling and biodistribution on RAJI tumoured mice. *J Nucl Med* **29**: 217–225

Fowler F (1990). Radiobiological aspects of low dose rates in radioimmunotherapy. *Int J Radiat Oncol Biol Phys* **18**: 1261–1269

Goldenberg DM, Sharkey RM and Ford E (1987) Anti-antibody enhancement of iodine-131 anti-CEA radioimmunodetection in experimental and clinical studies. *J Nucl Med* **28**: 1604–1610

Juweid M, Sharkey RM, Markowowitz A, Behr T, Swayne LC, Dunn R, Hansen HJ, Shevitz J, Leung SO, Rubin AD, Herskovic T, Hanley D and Goldenberg DM (1992) Treatment of non-Hodgkins lymphoma with radiolabelled murine, chimeric and humanised LL2 an anti-CD22 monoclonal antibody. *Cancer Res* **55**: 5144–5153

Keep PA, Searle F, Barratt GM, Boden J, Bagshawe KD and Rymann BE (1983) Clearance of injected radioactively labelled antibodies to tumour products by liposome-bound second antibodies. *Oncodev Biol Med* **4**: 273–280

King DJ, Turner A, Farnsworth APH, Adair JR, Owens RJ, Pedley RB, Baldock D, Proudfoot KA, Lawson ADG, Beeley NRA, Millar K, Millican A, Boyce BA, Antoniw P, Mountain A, Begent RHJ, Shochat D and Yarranton GT (1994) Improved tumour targeting with chemically cross-linked recombinant antibody fragments. *Cancer Res* **54**: 6176–6185

Lane DM, Eagle KF, Begent RHJ, Hope-Stone LD, Green AJ, Casey JL, Keep PA, Kelly AMB, Ledermann JA, Glaser MG and Hilson AJW (1994) Radioimmunotherapy of metastatic colorectal tumours with iodine-131-labelled antibody to carcinoembryonic antigen: phase I/II study with comparative biodistribution of intact and F(ab')$_2$ antibodies. *Br J Cancer* **70**: 521–525

Marshall D, Pedley RB, Boden JA, Boden R and Begent RHJ (1994) Clearance of circulating radioantibodies using streptavidin or second antibodies in a xenograft model. *Br J Cancer* **69**: 502–507

Marshall D, Pedley RB, Melton RG, Boden JA, Boden R and Begent RHJ (1995) Galactosylated streptavidin for improved clearance of biotinylated intact and F(ab')$_2$ fragments of an anti-tumour antibody. *Br J Cancer* **71**: 18–24

MIRD (Medical Internal Radiation Dose) (1975) Pamphlet number 11. Committee of the Society of Nuclear Medicine: USA

Norrgren K, Strand SE, Nilsson R, Lindgren L and Sjogen HO (1993) A general extracorporeal method to increase the tumour to normal tissue ratio in radioimmunoimaging and radioimmunotherapy. *J Nucl Med* **34**: 448–454

Pedley RB, Boden JA, Boden RW, Green A, Boxer GM and Bagshawe KD (1989) The effect of serum CEA on the distribution and clearance of anti-CEA antibody on the distribution and clearance of anti-CEA antibody in a pancreatic tumour xenograft model. *Br J Cancer* **60**: 549–554

Pedley RB, Boden JA, Boden R, Dale R and Begent RHJ (1993) Comparative radioimmunotherapy using intact or F(ab')$_2$ fragments of [illegible]I anti-CEA antibody in a colonic xenograft model. *Br J Cancer* **68**: 69–73

Press OW, Shan D, Howell-Clarke J, Eary J, Applebaum FR, Matthews D, King DJ, Haines AMR, Hamann P, Hinmann L, Shochat D and Bernstein ID (1996) Comparative metabolism and retention of Iodine-125, Yttrium-90, and Indium-111 radioimmunoconjugates by cancer cells. *Cancer Res* **56**: 2123–2129

Sharkey RM, Mabus J and Goldenberg DM (1988) Factor influencing anti-antibody enhancement of tumour targeting with antibodies in hamsters with human colonic tumour xenografts. *Cancer Res* **48**: 2005–2009

Sharkey RM, Juweid M, Shevitz J, Behr T, Dunn R, Swayne LC, Wong GY, Blumenthal RD, Griffiths GL, Siegal JA, Leung S, Hansen HJ and Goldenberg DM (1995) Evaluation of a complementary determining region grafted (humanised) anti-carcinoembryonic antigen monoclonal antibody in preclinical and clinical studies. *Cancer Res* **55**(suppl.): 5935–5945

Stephens S, Emtage S, Vetterlein O, Chaplin L, Bebbington C, Nesbitt A, Sopwith M, Athwal D, Novak C and Bodmer M (1995) Comprehensive pharmacokinetics of a humanised antibody and analysis of residual antiidiotypic responses. *Immunology* **85**: 668–674

Turner A, King DJ, Farnsworth APH, Rhind SK, Pedley RB, Boden J, Boden R, Millican TA, Millar K, Boyce B, Beeley NRA, Eaton MAW and Parker D (1994) Comparative biodistributions of indium-111 labelled macrocycle chimeric B72.3 antibody conjugates in tumour bearing mice. *Br J Cancer* **70**: 35–41

*© Cancer Research Campaign 1998*